# Longitudinal modelling of microbiome subcommunities reveals parity-dependent dynamics during pregnancy and postpartum

**DOI:** 10.1080/19490976.2026.2690907

**Published:** 2026-07-15

**Authors:** K. M. Kennedy, S. Fernando, S. A. Atkinson, M. G. Surette, P. Jeganathan, D. M. Sloboda

**Affiliations:** a Department of Biochemistry and Biomedical Sciences, McMaster University, Hamilton, Canada; b Farncombe Family Digestive Health Research Institute, McMaster University, Hamilton, Canada; c Department of Obstetrics and Gynecology, McMaster University, Hamilton, Canada; d Department of Mathematics and Statistics, McMaster University, Hamilton, Canada; e Department of Pediatrics, McMaster University, Hamilton, Canada; f Department of Medicine, McMaster University, Hamilton, Canada; g School of Computational Science and Engineering, McMaster University, Hamilton, Canada

**Keywords:** Microbiome, microbiota, microbial, metabolites, parity, pregnancy, perinatal, metabolism

## Abstract

**Background:**

Dysregulation of maternal adaptations to pregnancy due to high pre-pregnancy BMI (pBMI) is associated with worsened health outcomes for mothers and children. The role of the gut microbiome in these adaptations remains unclear.

**Methods:**

Stool samples were collected from pregnant participants (*n* = 52) enrolled in the Be Healthy in Pregnancy study (NCT01689961) during first, second, and third trimesters, and 6-months postpartum, along with samples from their infants at 6 months of age. Following 16S rRNA gene sequencing, we implemented time-aligned LDA (TALDA) using a time-weighted sampling strategy with exponentially decaying weights to construct time-proximal cohorts, applied LDA to each cohort independently, and aligned resulting topics using the alto R package. Infant samples were analyzed using standard LDA.

**Results:**

Seven distinct subcommunities were identified, one of which represented perineal contamination and was excluded from further analysis. Remaining subcommunities had distinct taxonomic definitions which remained stable throughout pregnancy (mean cross-cohort stability 0.899) while subcommunity proportions within individuals varied. Multiparous individuals showed greater shifts in subcommunity proportions during early pregnancy, while primiparous individuals displayed progressively increasing shifts with the largest changes during the transition from pregnancy to postpartum. High pBMI was associated with reduced microbiome remodelling, particularly among multiparous individuals. In exploratory analyses, maternal SCFA-producing subcommunity abundance at late pregnancy was positively associated with infant Bifidobacterium-dominated subcommunity proportions at 6 months, while pBMI > 25 was independently associated with lower infant Bifidobacterium.

**Conclusion:**

TALDA effectively distinguished between stable subcommunity definitions and dynamic subcommunity proportions, revealing that the microbiome maintains functional organization while subcommunity proportional contributions shift over the course of pregnancy and postpartum. These maternal dynamics may have intergenerational consequences through their influence on infant gut colonization. This work demonstrates that maternal factors modify microbiome trajectories and supports the existence of an ecological memory of pregnancy history within the maternal gut microbiome.

## Background

Maternal metabolic adaptations are essential to ensure appropriate maternal-fetoplacental energy partitioning during pregnancy. Dysregulation of these adaptations can lead to an increased risk of pregnancy complications and long-term metabolic dysfunction in both the mother and her child.^1^ These metabolic adaptations also persist well beyond the postpartum period.^2^ Accumulating evidence suggests that pregnancy-induced metabolic adaptations are influenced by the remodeling of the gut microbiome over the course of pregnancy.^3^ ^,^ ^4^ However, few studies have investigated how pregnancy history (parity) shapes microbiome dynamics over the course of pregnancy.^5^ ^,^ ^6^

The gut microbiota is characterized by high inter-individual variability.^7^ ^,^ ^8^ Different microbial taxa can occupy the same functional niche,^9^ making it difficult to identify functionally meaningful shifts in taxonomic composition. Traditional analytical methods focusing on diversity metrics or differential abundance of individual taxa do not fully capture the complexity of these communities.^10^ Previous efforts to identify interpretable groupings of microbial taxa have included clustering approaches such as those used to define enterotypes in the gut microbiota^11^ but have faced criticism for imposing discrete boundaries on a continuous gradient of taxonomic variation.^12^

Recent advances in statistical modeling, particularly Latent Dirichlet Allocation (LDA) topic modeling, have provided new approaches to identify subcommunities within microbiome data.^13^ Rather than classifying samples into discrete groups, LDA uses shared patterns of taxon co-occurrence to identify non-exclusive subcommunities, with each taxon having a probability of being associated with each subcommunity. Each sample can then be characterized by its proportions of these subcommunities, capturing the continuous nature of microbial variation. Previous microbiome studies have used this approach successfully to reveal insights into community structure and function that are not apparent when analyzing individual taxa in isolation.^14-17^

Microbiome communities change over time in response to numerous factors, including diet, health status, and environmental influences. However, most existing topic modeling approaches treat longitudinal samples as independent (e.g. traditional LDA) or as interchangeable repeated measures that account for within-subject correlation but do not model temporal dynamics (e.g. RM-LDA^18^). This limits the ability to characterize dynamic changes in developmental trajectories where progressive physiological changes occur over time, such as during pregnancy.

To address these limitations, we adapted a temporal cohort strategy from Wang et al.^19^ to track microbial subcommunities throughout pregnancy and postpartum. Rather than treating samples from different pregnancy stages as independent or assuming subcommunity composition is static across timepoints, we fit separate LDA models centered on each sampling timepoint, allowing subcommunity composition to vary over time. Because our sampling frequency is lower than in Wang et al.^19^, we replaced their shortest-path alignment algorithm with an optimal-transport approach implemented in the alto R package, which provides stable topic alignment with sparse temporal sampling. We refer to this procedure as time-aligned LDA (TALDA). TALDA allows us to distinguish between the taxonomic definition of a subcommunity and the proportion of that subcommunity within individual samples, both of which may change over the course of pregnancy and postpartum.

Previously, we demonstrated that pre-pregnancy BMI (pBMI) influences gut microbiome remodeling over the course of pregnancy, with differential effects based on parity status.^20^ Here, we implement TALDA to further characterize these dynamics. Based on our prior findings, we hypothesized that higher pBMI would be associated with reduced microbiome remodeling across pregnancy and that parity would modulate this effect, with multiparous individuals showing earlier microbiome shifts consistent with an “ecological memory” of prior pregnancy. Furthermore, because maternal microbial shifts are also associated with infant gut colonization, we explored potential relationships between maternal factors and infant gut microbiome composition at 6 months of age.

## Methods

### Study design and participants

Ethics approval was obtained from the Research Ethics Boards of Hamilton Health Sciences (REB Project#12-469), and Joseph Brant Hospital in Burlington, Canada (JBH 000-018-14). Pregnant participants recruited into the Be Healthy in Pregnancy (BHIP) study, a randomized controlled trial (RCT; Clinical Trials Ref: NCT01693510,^21^) were approached to enter the Bugs in BHIP sub-study to investigate gut microbiome trajectories. For the BHIP RCT, pregnant individuals were recruited at 12−17 weeks gestation, stratified by pre-pregnancy BMI (pBMI) category into 3 groups (18.5−24.9, 25–29.9, and 30–40), and randomized to an intervention or control group in a 1:1 allocation ratio. Full details of the BHIP RCT study protocol have been previously published.^22^ Briefly, participants randomized to the intervention group received a personalized nutrition plan with a high protein content (25% calories from protein) primarily from dairy sources and an exercise component consisting of a goal of 10,000 steps per day that includes 25–40-minute walking sessions 3−4 times per week. Inclusion criteria of the BHIP study^21^ included pBMI < 40; over 18 years of age; no diabetes or other known medical conditions; no contraindications to exercise; not opposed to consuming all food groups; and being a non-smoker.

### Sample collection

BHIP participants were evaluated at the end of the first (10–17 weeks), second (26–28 weeks), and third (>36 weeks) trimesters, and again at 6 months postpartum.^21^ At these visits, we obtained maternal stool samples that were self-collected by participants prior to each visit. Participants self-collected stool samples by wiping the perianal area with toilet paper immediately following defecation and placing the sample in provided collection bags. Samples were frozen at −20 °C immediately after collection by participants and transferred to −80 °C storage upon arrival at the laboratory until further use. At 6 months postpartum maternal and infant stool (in diapers) was collected and a questionnaire capturing infant dietary intake and supplement use was obtained to assess exposure to breastfeeding and solid food consumption, and supplemental probiotic use. Antenatal data was collected by BHIP from clinical charts, including mode of delivery, birth weight, and gestational age.^21^

### DNA extraction and amplification

Genomic DNA (gDNA) was extracted from stool samples as described previously^23^ with the addition of a mechanical lysis step using 0.2 g of 2.8 mm ceramic beads to improve extraction efficiency and without mutanolysin. Four negative controls were included on each extraction plate. PCR amplification of the variable 3 (V3) region of the 16S rRNA gene was performed on the extracted DNA using methods previously described.^24^ Each reaction contained 5 pmol of primer (341F, 518 R), 200 mM of dNTPs, 1.5 μl 50 mM MgCl2, 2 μl of 10 mg/ml bovine serum albumin (irradiated with a transilluminator to eliminate contaminating DNA) and 0.25 μl Taq polymerase (Life Technologies, Canada) for a total reaction volume of 50 μl. 341F and 518 R rRNA gene primers were modified to include adapter sequences specific to the Illumina technology and 6-base pair barcodes were used to allow multiplexing of samples. Lack of amplification in all negative controls was confirmed by gel electrophoresis and one negative control from each extraction plate was sequenced.

### Sequencing and data pre-processing

16S DNA products of PCR amplification were sequenced using the Illumina MiSeq platform (2x150bp) at the Farncombe Genomics Facility (McMaster University, Hamilton ON, Canada). Primers were trimmed from FASTQ files using Cutadapt^25^ (RRID: SCR_011841) and DADA2^26^ was used to derive amplicon sequence variants (ASVs). Taxonomy was assigned using the Silva 138 reference database.^27^ Non-bacterial ASVs were culled (kingdom Eukaryota, family Mitochondria, order Chloroplast, or no assigned phylum), as was any ASV to which only 1 sequence was assigned. To mitigate vaginal contamination of perianal swab samples, seven genera unambiguously associated with the vaginal/perineal microbiome (*Gardnerella*, *Anaerococcus*, *Peptoniphilus*, *Finegoldia*, *Fenollaria*, *Corynebacterium*, and *Lactobacillus*) were removed prior to topic modeling. All samples retained sufficient sequencing depth following filtering (>5000 reads).

### Time-aligned topic modeling approach

#### Topic number determination

To determine the optimal number of topics for the TALDA model, we implemented a multi-criteria approach that balanced topic coherence with model stability. ASVs were aggregated at the genus level (or lowest classified rank) using tax_glom in phyloseq. Using the alto R package,^28^ we fit LDA models on all samples with topic numbers ranging from 1 to 20 and aligned resulting models using the transport method. Raw (unnormalized) count data were used as rarefaction or normalization to relative abundances would violate the model's generative assumption that each sequencing read is an independent draw from a multinomial distribution over taxa. We evaluated models based on minimum topic coherence, alignment topology, and biological interpretability.

#### Time-aligned LDA framework

To address the temporal nature of microbiome samples collected across pregnancy and postpartum periods, we adapted a longitudinal topic modeling framework that extends traditional LDA to incorporate temporal structure. In our approach, the optimal-transport alignment performs a global matching of all topics across consecutive time windows by minimizing total compositional discrepancy, yielding stable and coherent topic trajectories that better reflect temporal transitions. TALDA accounts for the sequential nature of longitudinal samples rather than treating them as independent observations. We implemented a time-weighted sampling strategy (described in^28^) to generate one cohort for each sampling time point in our study, resulting in four cohorts. We selected this number of cohorts to reflect the discrete and relatively sparse sampling design; unlike streaming data with thousands of time points, pregnancy microbiome studies typically include only a few clinically defined collection times, and the cohort structure was chosen to respect this temporal granularity. For each cohort, samples from the focal timepoint were given full weight (1.0), while samples from other time points were assigned exponentially decaying weights based on their temporal distance from the focal point. These weights were then normalized to ensure proper sampling proportions while maintaining the focal time point at full representation. This approach created a “temporal window” around each time point that incorporated information from nearby time points with decreased influence as temporal distance increased. Specifically, for a given focal time point t, the weight assigned to samples from time point s was: • 1.0 if t = s • *λ* ^|t-s|/U for t ≠ s where *λ* = 0.75, |t-s| is the temporal distance between time points t and s, and U is a normalization factor (the sum of all non-focal weights). For each time-weighted cohort, we fit a separate LDA topic model with K = 7 topics, determined as described above (Topic number determination). Because the exponential weighting scheme produces overlapping cohorts, many samples contribute to more than one cohort, though with different weights. These overlapping samples provide shared information across consecutive cohorts, enabling more stable alignment of topics over time. Importantly, while cohorts overlap for modeling purposes, the topic proportions for each sample reflect the time point at which the sample was originally collected. The resulting four cohort-specific LDA models, each emphasizing a different temporal window, were then aligned using an optimal-transport procedure adapted from alto, repurposed here to align topics across time rather than across model sizes.

### Model validation and sensitivity analyzes

To assess whether TALDA topics represent consistent biological subcommunities rather than artifacts of the time-weighted cohort structure, we compared TALDA outputs against a standard LDA model fit across all samples simultaneously. The standard LDA model was fit using the alto R package with identical preprocessing to the TALDA pipeline. Agreement was evaluated for both topic proportions per sample (gamma) using Spearman rank correlation, and taxon weights per topic using cosine similarity.

The exponential decay parameter (*λ* = 0.75) was selected to provide moderate down-weighting of temporally distant samples while retaining information from non-adjacent timepoints. To assess robustness to this choice, we compared TALDA solutions across three decay values: *λ* = 0.5, 0.75, and 0.9. Topic composition (beta matrices) was highly stable across all values (mean cosine similarity > 0.96; Table S3).

### Statistical analysis

#### Participant characteristics

Participant demographic and clinical characteristics were summarized stratified by parity status (primiparous vs. multiparous) and pre-pregnancy BMI category (<25kg/m2 vs. ≥25kg/m2 according to WHO definition of overweight).^29^ All analyzes were conducted using the gtsummary package^30^ (v2.0.0; RRID: SCR_021319) in R.

#### Microbiome analysis

Subcommunity compositional stability across pregnancy was assessed by calculating Jensen–Shannon divergence between the taxonomic composition of each subcommunity at adjacent time points. Divergence values were converted to stability scores (1—JS divergence),^31^ with values closer to 1 indicating greater compositional subcommunity similarity between time points. Stability scores were summarized (mean value) across transitions and visualized to identify patterns of temporal stability.

To characterize how subcommunity proportions varied across participant groups and pregnancy stages, we calculated 95% confidence intervals for the mean topic proportions of each subcommunity stratified by pBMI category (<25 vs. ≥25), parity (primiparous vs. multiparous), and time point (T1, T2, T3, PP). Confidence intervals were estimated using percentile bootstrap methods (2,000 iterations) to account for the constrained nature of compositional data, where topic proportions sum to 1 within each sample. These intervals are descriptive summaries intended to visualize group-level patterns and were resampled at the sample level; within-subject dependence was addressed in subsequent mixed-effects analyzes. Non-overlapping confidence intervals were interpreted as suggestive of meaningful differences between groups. Analyzes were conducted using the compositions package (v2.0-8) and boot package (v1.3-31) in R.

#### Temporal dynamics analysis

To quantify within-participant changes in subcommunity proportions over time, Euclidean distances were calculated between topic proportion vectors for each participant across three temporal transitions: first to second trimester (T1-T2), second to third trimester (T2-T3), and third trimester to 6 months postpartum (T3-PP).

We fit a linear mixed-effects model using the lme4^32^ and lmerTest R packages^33^ with Euclidean distance as the outcome. Fixed effects included pBMI category (<25 vs. ≥25), parity (primiparous vs. multiparous), temporal transition (T1-T2, T2-T3, T3-PP), and interactions for pBMI x parity and parity x transition. A random intercept for participant ID accounted for repeated measures within individuals. Effects were assessed using Type III analysis of variance with Satterthwaite approximation for denominator degrees of freedom. We performed post-hoc comparisons of estimated marginal means using the emmeans R package^34^ with Benjamini-Hochberg correction for multiple testing.

To assess inter-individual variability in temporal trajectories, we constructed a participant-by-transition matrix of Euclidean distances (T1-T2, T2-T3, T3-PP). Missing transition values were imputed with column means. We applied hierarchical clustering (with Ward's D2 linkage method) to the resulting inter-participant distance matrix, with the optimal number of clusters determined by silhouette analysis. Principal component analysis was performed on the transition matrix to visualize trajectory patterns in reduced dimensions. Associations between trajectory cluster membership and participant characteristics (pBMI category, parity, intervention group) were tested using chi-squared tests.

#### Infant microbiome analysis

Infant stool samples collected at 6 months postpartum were analyzed separately using traditional LDA to identify subcommunities within the infant gut microbiome. Because infant samples represented a single cross-sectional time point, time-aligned methods were not applicable. We applied the model selection procedure described above in the topic number determination methods subsection.

Due to the known influence of probiotic supplementation on infant gut microbiome composition, infants with reported probiotic use at 6 months (*n* = 9) were excluded from analyzes examining associations with maternal factors. We calculated 95% confidence intervals for the mean topic proportions of each subcommunity stratified by maternal parity, maternal pre-pregnancy BMI, delivery mode (vaginal vs. Cesarean section), and solid food introduction status. Confidence intervals were estimated using percentile bootstrap methods (2,000 iterations) as described above for maternal samples. Given the limited sample size and multiple stratification factors, these analyzes were considered exploratory.

#### Data and code availability

Sequencing data have been deposited at the NCBI Sequence Read Archive (SRA; PRJNA1404870) and are publicly available as of the date of publication. All original code has been deposited on GitHub (https://github.com/kennek6/Kennedy2026_TALDA) and is publicly available as of the date of publication. Any additional information required to reanalyze the data reported in this paper is available from the corresponding authors upon request.

## Results

### Participant characteristics

A total of 65 participants were recruited, and stool samples were collected during the first trimester (T1), second trimester (T2), and third trimester (T3) of pregnancy as well as at 6 months postpartum (PP). After excluding participants with fewer than 2 pregnancy samples collected, maternal samples from 52 individuals were analyzed (T1 *n* = 45, T2 *n* = 48, T3 *n* = 43, PP, *n* = 43). The majority of participants delivered vaginally (37/52; 71%) and rates of vaginal delivery did not differ by parity (primiparous *n* = 32, multiparous *n* = 20; *p* = 0.11, Pearson's Chi-squared test) or pre-pregnancy BMI category (pBMI < 25 *n* = 35, ≥ 25 *n* = 17; *p* = 0.7). Multiparous participants were older than primiparous participants (*p* = 0.001), but there was no significant effect of parity or pBMI on gestational weight gain (GWG) or length of gestation (Table 1). There was no significant difference in parity, pBMI, maternal age, GWG, length of gestation, delivery mode, infant sex, infant birth weight, or birth length between participants assigned to the intervention or control groups in the subset of participants included in this study (Supplementary Table 1) or the overall BHIP study cohort.^19^

**Table 1.** Maternal characteristics.

	Overall by Parity			Overall by pBMI		
Characteristic	primiparous	multiparous	*p*	<25	≥25	*p*
**n**	32	20		35	17	
**Primigravida**	26 (81%)	0 (0%)	**<0.001^3^**	16 (46%)	10 (59%)	0.4^2^
**Age**	31.4 (3.9)	34.8 (3.9)	**0.001^1^**	33 (4.5)	32.2 (3.3)	0.7^1^
**pBMI**	23.5 (3.9)	24.7 (4.6)	0.5^1^	21.6 (1.8)	28.8 (3.6)	**<0.001^1^**
**GWG (kg/week)**	0.47 (0.13)	0.49 (0.16)	0.5^1^	0.46 (0.13)	0.5 (0.17)	0.1^1^
**Length of gestation (days)**	280 (7)	278 (7)	0.4^1^	280 (6)	279 (10)	0.7^1^
**Delivery mode = vaginal**	20 (65%)	17 (85%)	0.1^2^	26 (74%)	11 (69%)	0.7^3^

Mean (SD); n (%).

^1^ Wilcoxon rank sum exact test.

^2^ Pearson's Chi-squared test.

^3^ Fisher's exact test.

### TALDA identifies seven microbial subcommunities with distinct temporal patterns

**Figure 1.**
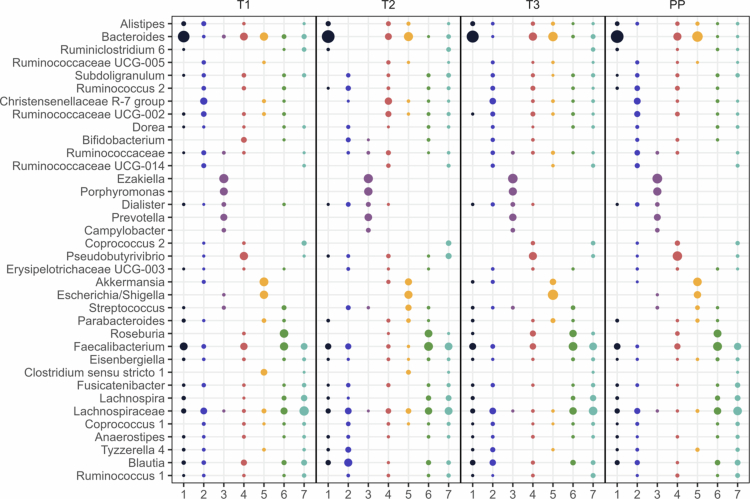
Taxonomic composition of topics across time-aligned cohorts. Relative contributions of bacterial genera to each of the seven TALDA-defined subcommunities (topics) across four time-weighted cohorts centered on first trimester (T1), second trimester (T2), third trimester (T3), and postpartum (PP). Dot size corresponds to posterior topic–taxon probabilities (*β*) for K = 7 subcommunities, with colors indicating individual subcommunities. Within each panel, genera with β ≥ 0.005 are shown and ordered by their relative contributions.

TALDA identified seven subcommunities with distinct microbial signatures. Each subcommunity (SC) was characterized by a unique taxonomic profile that showed varying patterns across the time-weighted cohorts (Figure 1). SC1 was strongly dominated by *Bacteroides*, with secondary contributions from *Faecalibacterium*, *Blautia*, and *Alistipes*. SC5 was also *Bacteroides*-enriched but more evenly characterized by co-dominance with *Escherichia/Shigella* and *Akkermansia*. SC6 was dominated by canonical butyrate-producing taxa (*Faecalibacterium*, *Roseburia*, and Lachnospiraceae) while SC7 shared many of these taxa but distributed more evenly across a broader range of Lachnospiraceae members including *Lachnospira*, *Coprococcus*, and *Fusicatenibacter*, suggesting a more diverse fermentative community. SC4 was distinguished by *Pseudobutyrivibrio* and *Bacteroides* co-dominance, with smaller, variable contributions from *Ruminococcaceae* and *Lachnospiraceae* members. SC2 was the most taxonomically diffuse, with relatively even contributions from Lachnospiraceae, *Blautia*, *Christensenellaceae* R–7 group, and multiple *Ruminococcaceae* members. While SCFA-producing taxa were present across most subcommunities, their relative dominance and co-occurrence partners varied, reflecting distinct community configurations rather than a single fermentative archetype. SC3 was taxonomically distinct, dominated by taxa prevalent in the vaginal niche including *Ezakiella*, *Porphyromonas*, and *Prevotella*. As samples were collected perianally, this subcommunity likely reflects residual perineal contamination not captured by our conservative taxonomic filtering and was therefore excluded from downstream analyzes. The segregation of these taxa into a single subcommunity rather than being distributed across all subcommunities supports the ability of our modeling approach to distinguish biologically distinct signals within the data.

**Figure 2.**
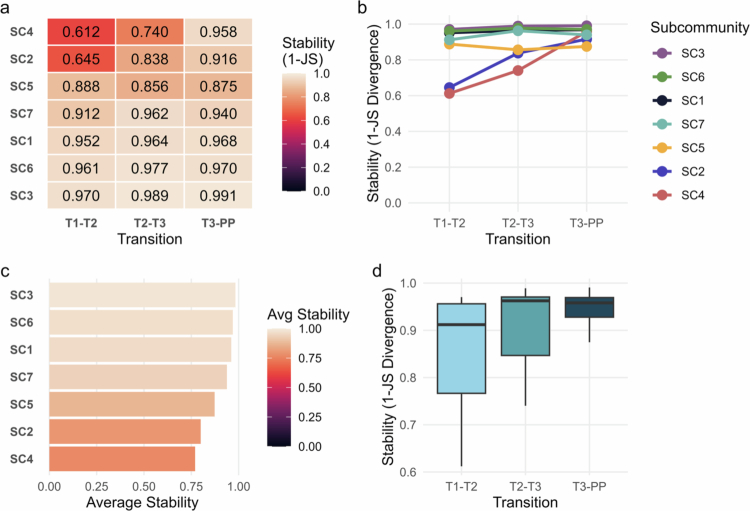
Subcommunity stability across pregnancy and postpartum. Stability of subcommunity taxonomic profiles was assessed using Jensen–Shannon (JS) divergence, expressed as stability scores (1—JS divergence). (a) Heatmap showing stability scores for each subcommunity across consecutive transitions (T1–T2, T2–T3, T3–PP). Higher values indicate greater similarity in subcommunity composition between time points. (b) Line plots of stability trajectories for each subcommunity, with colors indicating the seven topics. (c) Average stability of each subcommunity across all transitions, with bar length and color representing mean stability values. (d) Distribution of stability scores across all subcommunities, shown as boxplots for each transition (median and interquartile range (IQR); whiskers extend to 1.5 × IQR).

We next asked whether the taxonomic definitions of these subcommunities are stable across pregnancy and postpartum, or whether specific taxa at particular transitions introduce instability. To assess this, we calculated Jensen-Shannon divergence across transitions, converted to stability scores (1 - JS divergence; Figure 2).^25^ Subcommunities 1, 6, and 7 showed the highest average stability (>0.9), maintaining consistent composition throughout pregnancy and postpartum. This stability is consistent with core functional niches (e.g. SCFA production) and? are robust to pregnancy-associated shifts. SC5 has slightly lower stability overall (>0.85), driven by increasing contributions from *Escherichia*/*Shigella* (0.20 to 0.33) and decreasing contributions from *Akkermansia* (0.23 to 0.10) and *Clostridium sensu stricto* (0.11 to < 0.001) over the course of pregnancy. Subcommunities 2 and 4 both showed patterns of increasing stability over time, with the first-to-second trimester transition (T1-T2) being the least stable. In SC2, this early instability was driven by notable fluctuations among its defining taxa: *Christensenellaceae R-7 group* contributed substantially in the first trimester (0.13) but dropped markedly in the second (0.006) before partially recovering in the third trimester (0.10), while Blautia showed the opposite pattern, increasing from 0.03 in the first trimester to 0.18 in the second before returning to 0.10 by the third. A similar pattern was observed for *Ruminococcaceae UCG-002* and *Faecalibacterium*. This oscillatory reshuffling among taxonomically diverse Firmicutes members is consistent with functional redundancy, with multiple fiber-fermenting and SCFA-producing taxa possibly competing for representation within the subcommunity definition during early pregnancy, before settling into a more stable configuration by the third trimester and postpartum. SC4 showed a similar trajectory of increasing stability, though the T1-T2 instability reflected large shifts among secondary contributors: *Christensenellaceae R-7 group* was nearly absent in the first trimester (<0.001) but surged to 0.14 in the second before declining again, while Faecalibacterium decreased from 0.14 in the first trimester to 0.03 in the second. These reciprocal shifts among secondary taxa, while the dominant contributors remained relatively stable, suggest that SC4's core identity was maintained even as the broader ecological context of the subcommunity was reorganizing during early pregnancy. These taxon-level dynamics, which are not detectable by standard LDA, demonstrate the additional resolution provided by the time-aligned approach.

### Maternal factors shape subcommunity proportions

**Figure 3.**
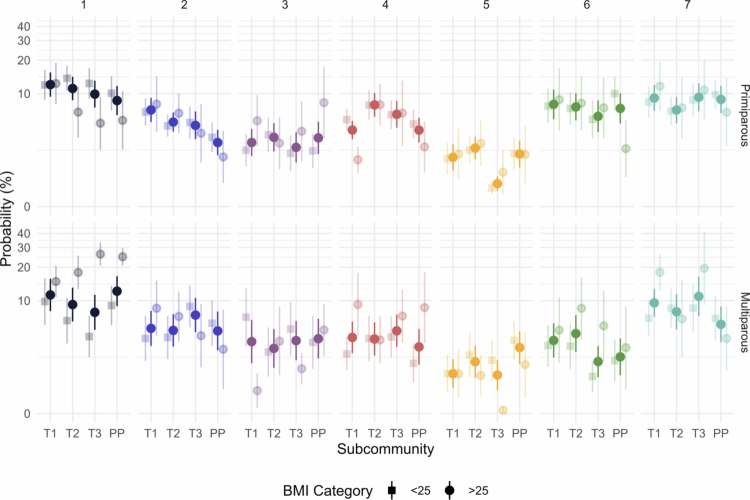
Parity and pre-pregnancy BMI impact subcommunity proportion probabilities across pregnancy and postpartum. Mean (points) ± s.e.m. (error bars) of subcommunity (topic) proportions across pregnancy and postpartum. Panels are arranged by parity (rows: Primiparous, Multiparous) and sampling time (columns: T1, T2, T3, PP). Semi-transparent points/error bars show BMI-specific means (pBMI < 25 - ■, ≥ 25 - ●), overlaid with opaque points/error bars indicating the panel mean across BMI categories for each subcommunity.

We next assessed how subcommunity proportions within participants varied over time and their associations with host factors. Bootstrap confidence intervals were calculated to visualize patterns in subcommunity proportions across groups; non-overlapping intervals were interpreted as suggestive of meaningful differences. Distinct patterns of subcommunity abundances across pregnancy and postpartum periods were associated with both pre-pregnancy BMI and maternal parity (Figure 3).

Despite the reshuffling of secondary contributors during early pregnancy, SC4 maintained its core taxonomic identity (Pseudobutyrivibrio and Bacteroides dominance) across timepoints. Its relative abundance showed temporal dynamics that differed by parity and pBMI. Among primiparous individuals, proportions increased from first to second trimester (T1: 0.07 [0.05–0.10]; T2: 0.16 [0.10–0.22]), though this increase began from different starting points depending on pBMI category. In the first trimester, primiparous individuals with pBMI < 25 had higher proportions (0.08 [0.06–0.12]) than those with pBMI ≥ 25 (0.03 [0.01–0.05]), but by the second trimester those with pBMI ≥ 25 had increased (0.14 [0.06–0.24]). Multiparous individuals showed no significant temporal change in SC4 proportions regardless of BMI category.

The relationship between pBMI and SC1 (*Bacteroides*-dominated) proportions varied by parity. Overall proportions appeared similar between BMI groups (pBMI < 25: 0.21 [0.17–0.25]; pBMI ≥ 25: 0.19 [0.15–0.24]), but this masked opposing trends within parity groups. Among primiparous individuals, pBMI < 25 tended towards higher SC1 proportions (0.22 [0.18–0.27] vs. 0.15 [0.09–0.21]) while among multiparous individuals, pBMI ≥ 25 tended towards higher proportions (0.24 [0.18–0.31] vs. 0.18 [0.12–0.25]). Although confidence intervals overlapped in all comparisons, this divergent pattern was consistent across timepoints and appeared to strengthen over pregnancy, with the greatest log2 fold changes in the third trimester in both primiparous (log2FC = −1.38; pBMI < 25: 0.24 [0.13–0.35]; pBMI ≥ 25: 0.11 [0.03–0.21]) and multiparous individuals (log2FC = 1.11; pBMI < 25: 0.11 [0.04–0.21; pBMI ≥ 25: 0.30 [0.18–0.44]).

SC6 (*Faecalibacterium*, *Roseburia*, Lachnospiraceae) showed the most consistent difference by parity, with higher proportions in primiparous individuals (0.19 [95% CI 0.14–0.23]) compared to multiparous individuals (0.10 [0.07–0.13]) across all timepoints. Unlike SC4, this difference did not vary over the course of pregnancy: primiparous individuals maintained higher proportions from the first trimester through postpartum, with overlapping confidence intervals at individual timepoints but a consistent directional separation. This parity effect was most apparent among those with pBMI < 25 (primiparous: 0.19 [0.14–0.25]; multiparous: 0.08 [0.05–0.11]).

While these patterns suggest associations between maternal factors and subcommunity proportions, the high degree of interindividual variability in microbiome composition limited our ability to detect consistent group-level differences. Individual participants showed substantial heterogeneity in their subcommunity profiles even within the same parity and BMI categories, with confidence intervals frequently overlapping across groups. To complement these group-level patterns and account for interindividual variation, we next quantified within-participant shifts across pregnancy transitions.

### Within-participant microbiome dynamics reveal BMI and parity effects

**Figure 4.**
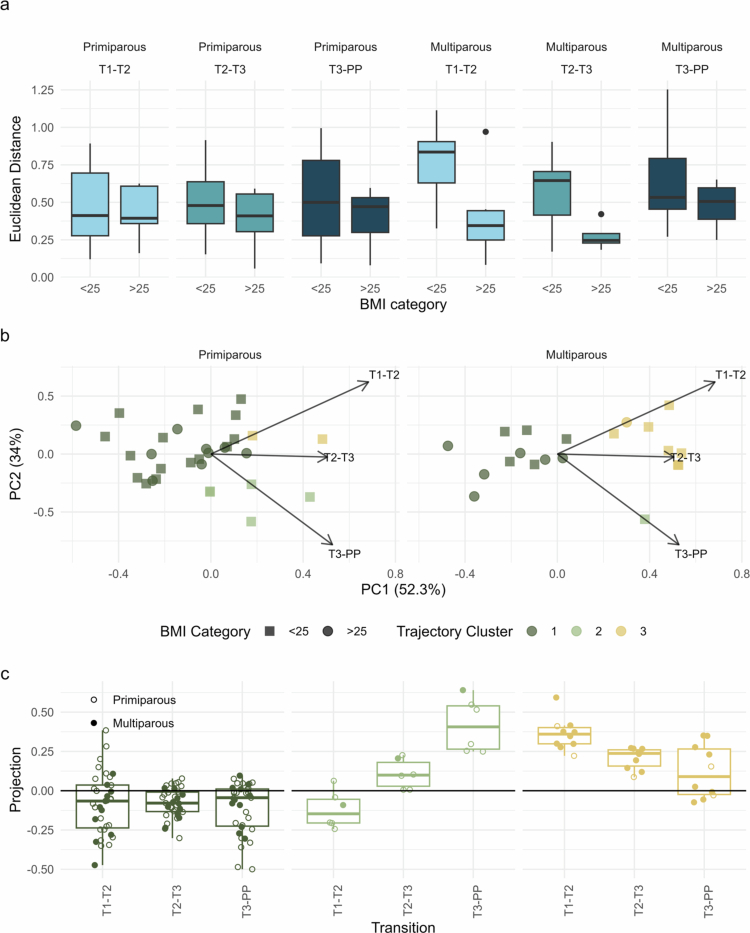
Host factors shape longitudinal microbiome trajectories. (a) Within-participant Euclidean distances of subcommunity proportions between consecutive time points, shown by BMI category (<25, ≥25). Boxplots display median and interquartile range (IQR); whiskers extend to 1.5 × IQR. (b) Principal component analysis (PCA) of participant trajectory patterns derived from Euclidean distances across transitions. Points are colored by trajectory cluster and shaped by BMI category; panels are split by parity. Arrows denote loading vectors for transition contrasts (T1–T2, T2–T3, T3–PP), indicating the primary directions contributing to separation in the PCA space. (c) Projections of each participant’s trajectory onto the loading vectors in (b), stratified by trajectory cluster. Boxplots display median and interquartile range (IQR); whiskers extend to 1.5 × IQR.

To assess how pre-pregnancy BMI and parity influence the degree of microbiome change across pregnancy, we calculated Euclidean distances between subcommunity proportion vectors for each participant across three temporal transitions (Figure 4). SC3 was excluded from this analysis as it was dominated by vaginal taxa. Within-participant distances show significant effects (linear mixed-effects model) of pBMI on microbiome stability (Figure 4a). Euclidean distances between samples within individuals were higher in participants with a pBMI < 25 compared to those with a pBMI ≥ 25 (F = 7.08, *p* = 0.011), indicating larger shifts in subcommunity proportion probabilities over the course of pregnancy and postpartum. Post-hoc comparisons revealed that this pBMI effect was driven by multiparous participants, among whom pBMI ≥ 25 was associated with lower Euclidean distances (*p* = 0.011), while no significant pBMI effect was observed within primiparous participants (*p* = 0.34). During the first-to-second trimester transition, multiparous participants showed greater shifts than primiparous women (*p* = 0.048), and this difference was not present during later transitions (*p* > 0.29).

Individual microbiome trajectories were determined by performing principal component analysis on within-participant Euclidean distances across temporal transitions (Figure 4b). Trajectory cluster membership was significantly associated with both parity (χ² = 9.15, *p* = 0.010) and pBMI (χ² = 7.01, *p* = 0.030). Primiparous individuals are more likely to have trajectories characterized by increasing microbial shifts throughout pregnancy with the greatest difference during the T3-PP transition (cluster 2) compared to multiparous individuals (Figure 4b-c). In contrast, multiparous individuals are more likely to have trajectories characterized by the greatest shifts early in pregnancy (T1-T2 transition, cluster 3). Participants with pBMI ≥ 25 were predominantly assigned to cluster 1, characterized by minimal shifts across all transitions (15/16, 94%), whereas participants with pBMI < 25 were distributed across all three trajectory clusters.

### Maternal parity and pBMI associate with infant microbiome composition at 6 months

Of the 52 participants included in our study, 46 provided a matched infant stool sample at 6 months of age. Most of these infants were vaginally delivered (34 of 46; 74%), breastfed (41 of 46; 89%), and eating solid food (39 of 46; 85%) at the time of sampling. There was no significant effect of maternal parity, pBMI, or infant sex on length of gestation, delivery mode, birth weight or length, or rates of breastfeeding, solid food consumption, or probiotic use at 6 months of age (Table 2).

**Table 2.** Infant characteristics and dietary exposures at 6 months of age.

	Parity			pBMI			Sex		
Characteristic	primiparous	multiparous	*p*	<25	≥25	*p*	female	male	*p*
**n**	28	18		32	14		23	23	
**Length gestation (days)**	281.4 (5.8)	278.9 (6.5)	0.2^1^	279.5 (6.1)	282.4 (6)	0.2^1^	281.6 (6)	279.3 (6.2)	0.2^1^
**Vaginal delivery**	18 (67%)	15 (83%)	0.3^3^	24 (75%)	9 (69%)	0.7^3^	15 (65%)	18 (82%)	0.2^2^
**Birth weight (g)**	3567 (292)	3594 (378)	0.8^1^	3564 (347)	3607 (273)	0.6^1^	3507 (322)	3648 (318)	0.1^1^
**Birth length (cm)**	52.3 (2.6)	51.3 (1.9)	0.4^1^	52.0 (2.1)	51.4 (2.9)	0.2^1^	51.7 (2.6)	52.0 (2.1)	0.8^1^
**Breastfed at 6 months**	26 (93%)	15 (83%)	0.4^3^	29 (91%)	12 (86%)	0.6^3^	21 (91%)	20 (87%)	1.0^3^
**Solid food at 6 months**	24 (86%)	15 (83%)	1.0^3^	27 (84%)	12 (86%)	1.0^3^	21 (91%)	18 (78%)	0.4^3^
**Probiotics at 6 months**	7 (25%)	2 (11%)	0.4^3^	7 (22%)	2 (14%)	0.7^3^	3 (13%)	6 (26%)	0.5^3^

Mean (SD); n (%).

^1^ Wilcoxon rank sum exact test.

^2^ Pearson's Chi-squared test.

^3^ Fisher's exact test.

**Figure 5.**
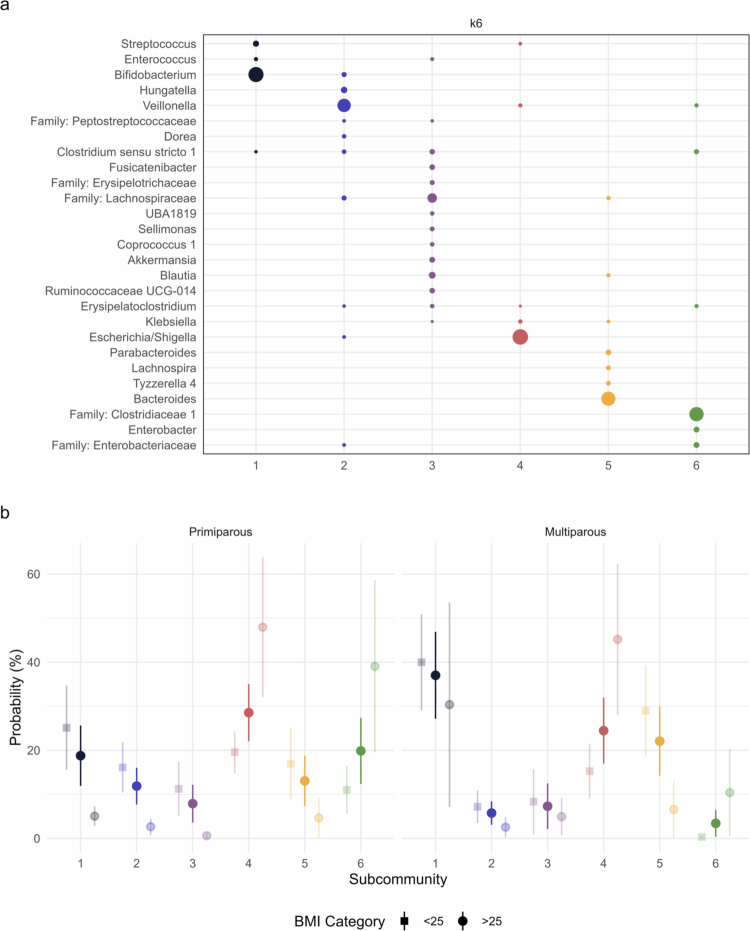
Maternal factors influence infant gut subcommunity composition at 6 months of age. (a) Taxonomic composition of subcommunities (topics) in infant gut microbiomes at 6 months of age. Dot size corresponds to posterior topic–taxon probabilities (*β*) for K = 6 subcommunities, with colors indicating individual subcommunities. Genera with β ≥ 0.005 are shown and ordered by their relative contributions to each subcommunity. (b) Maternal parity and pre-pregnancy BMI impact infant subcommunity proportion probabilities at 6 months. Mean (points) ± s.e.m. (error bars) of subcommunity (topic) probabilities stratified by maternal parity (Primiparous, Multiparous). Semi-transparent points/error bars (■, ●) show BMI-specific means (pBMI < 25, ≥ 25), overlaid with opaque points/error bars indicating the panel mean across BMI categories for each subcommunity.

Traditional LDA topic modeling identified six infant gut subcommunities, dominated by *Bifidobacterium* (SC1), *Veillonella* (SC2), *Lachnospiraceae* (SC3), *Escherichia*/*Shigella* (SC4), *Bacteroides* (SC5), and Clostridiaceae (SC6), respectively (Figure 5a). Due to the known association between breastfeeding and the composition of the infant gut microbiome^35^ and the limited number of infants exclusively fed formula in our study (*n* = 5), we assessed the impacts of delivery mode, solid food introduction, and probiotic intake only in infants who were breastfed (Fig. S1). Among breastfed infants not consuming probiotics, those eating solid foods had a higher mean proportion of SC6 (0.16 [0.06–0.28] vs. no solids: 0.0001 [0.000002–0.0003]) and a lower mean proportion of SC1 (0.21 [0.12–0.32] vs. no solids: 0.50 [0.17–0.82]). Conversely, among breastfed infants consuming solids, probiotic use was associated with a higher mean proportion of SC1 (0.51 [0.28–0.73] vs. no probiotics: 0.21 [0.12–0.32]) and a lower mean proportion of SC6 (0.004 [0.001–0.007] vs. no probiotics: 0.16 [0.06–0.28]). Among breastfed infants eating solids, delivery by Cesarean section (CS) was associated with a near complete absence of SC5 (0.001 [0.0002–0.001] vs. vaginal: 0.19 [0.10–0.30]) regardless of probiotic consumption. Infants with reported probiotic use at 6 months of age and those who were not breastfed were excluded from further analyzes.

Maternal parity and pre-pregnancy BMI were also associated with compositional shifts in the infant microbiota (Figure 5b). Infants born to mothers with pBMI ≥ 25 had a higher mean proportion of SC4 (0.47 [0.27–0.66] vs. pBMI < 25: 0.18 [0.11–0.25]) and a lower mean proportion of SC2 (0.03 [0.004–0.05] vs. pBMI < 25: 0.12 [0.06–0.20]). Infants born to mothers with pBMI ≥ 25 also tended toward lower proportions of SC5 (0.05 [0.0005–0.13] vs. pBMI < 25: 0.22 [0.10–0.35]), though confidence intervals overlapped. Among infants of mothers with pBMI < 25, those born to multiparous mothers had lower proportions of SC6 (0.003 [0.001–0.007]) compared to primiparous mothers (0.11 [0.01–0.23]). SC1 showed modest associations with both parity and pBMI, with higher proportions among infants of multiparous mothers (0.37 [0.19–0.56] vs. primiparous: 0.19 [0.08–0.33]) and among those with maternal pBMI < 25 (0.31 [0.18–0.45] vs. pBMI ≥ 25: 0.15 [0.03–0.35]), though confidence intervals overlapped in both comparisons.

To investigate associations between maternal late-pregnancy (T3) gut microbiota and infant gut microbiota at 6 months, we focused on a strictly defined subset of vaginally delivered, breastfed mother-infant dyads who were not supplemented with probiotics (*n* = 23). Using log-log linear regression adjusted for pBMI and parity, the proportion of maternal SC7 at T3 was positively and strongly associated with the proportion of infant SC1 at 6 months (*β* = 0.73, *p* = 0.0006, FDR = 0.02). Taxonomically, maternal SC7 was dominated by short-chain fatty acid (SCFA) producers, including *Lachnospiraceae*, *Faecalibacterium*, and *Lachnospira*. In contrast, infant SC1 was dominated by *Bifidobacterium.* Notably, these two associated subcommunities shared no dominant taxa, suggesting an indirect ecological link rather than direct vertical transmission of specific microbes from mother to infant. Furthermore, within this regression model, maternal pBMI ≥ 25 emerged as an independent predictor of lower infant SC1 proportions (*β* = −1.31, *p* = 0.018), confirming the initial suggestive trends observed in our broader compositional analysis.

## Discussion

Maternal metabolic adaptations to pregnancy may be mediated, in part, by shifts in the gut microbiome. In this study, we applied a longitudinal topic modeling approach to characterize microbial subcommunity dynamics over the course of pregnancy and postpartum and to investigate how pre-pregnancy BMI (pBMI) and maternal parity influence these trajectories. Overall, we found that the taxonomic definitions of microbial subcommunities remained stable throughout pregnancy and postpartum while proportions of these subcommunities within individuals changed, suggesting that pregnancy may represent a rebalancing among existing maternal microbial communities rather than dysbiotic disruption. Higher pBMI was associated with less microbiome remodeling, particularly in multiparous participants. The timing of maximal microbiome remodeling varied by maternal parity, with multiparous mothers experiencing greater shifts from first to second trimester while primiparous mothers experienced the greatest shifts during the postpartum transition. These patterns suggest the microbiome retains an “ecological memory” of prior pregnancies, which could enable more rapid adaptation in subsequent pregnancies.

The stability of subcommunity definitions was not uniform. Subcommunities 2 and 4 showed the greatest compositional instability during early pregnancy, but this instability was driven by reshuffling among functionally similar secondary contributors rather than loss of core taxa. In SC2, multiple Firmicutes fiber-fermenters (*Christensenellaceae* R-7 group, *Blautia*, *Ruminococcaceae UCG-002*) showed reciprocal oscillations across the first and second trimesters before stabilizing. This is consistent with functional redundancy within these subcommunities: multiple taxa capable of occupying similar metabolic niches compete for representation during a period of ecological disruption, without altering the subcommunity's overall functional capacity. That these subcommunities are taxonomically diffuse yet functionally coherent suggests they represent real ecological units—diverse guilds with interchangeable members—rather than statistical artifacts of the modeling process. Whether compositional turnover within these subcommunities corresponds to functional stability (i.e., maintained SCFA production despite shifting producers) remains to be tested with metagenomic or metabolomic data.

Parity emerged as a modulator of microbiome dynamics, with distinct signatures across subcommunities. SC4 showed temporal dynamics only among primiparous individuals, whose proportions increased from first to second trimester while multiparous individuals showed no change. SC6 was higher in primiparous individuals across all timepoints. These represent two different signatures of parity's influence: SC4 reflects differential responsiveness to pregnancy (primiparous microbiomes reorganize, multiparous do not), while SC6 reflects a lasting compositional shift associated with prior pregnancy. Together, they suggest that the microbiome retains an ecological memory of prior pregnancies that operates through at least two mechanisms—buffering against perturbation and persistent alteration of community structure. This is consistent with our previous observations in a German pregnancy cohort^20^ and extends them by identifying the specific subcommunities through which parity effects manifest.

The mechanism underlying this ecological memory may operate through host, microbial, or joint pathways. From the host perspective, prior pregnancy exposure may prime immune adaptations—including memory T cell populations shaped by prior microbial antigen exposure—that constrain or direct microbiome reorganization in subsequent pregnancies. From a microbial perspective, communities that have previously undergone pregnancy-associated restructuring may harbor taxa or ecological configurations that facilitate more efficient adaptation upon re-exposure to the same hormonal or metabolic milieu. These possibilities are not mutually exclusive; iterative host-microbe co-adaptation across pregnancies may progressively stabilize the system's response trajectory. Distinguishing these mechanisms will require longitudinal studies with immune profiling and pre-pregnancy microbiome characterization across multiple gestations.

Differences in the timing of microbiome remodeling due to maternal parity may influence infant gut microbiome assembly. At 6 months of age, we found that the microbiome of infants born to multiparous mothers had higher proportions of a *Bifidobacterium*-dominated SC1. As we previously found that *Bifidobacterium* abundance is higher in multiparous mothers compared to primiparous mothers,^20^ this may reflect increased mother-to-infant transmission of this genus. Interestingly, the observed lower proportions of this subcommunity with maternal pBMI ≥ 25 (vs. < 25) were particularly evident among primiparous infants, as were higher proportions of a Clostridiaceae-dominated SC6. It appears that later-born infants (those born to multiparous mothers) are protected from some of the effects of high maternal pBMI observed among first-born infants (born to primiparous mothers). These data are consistent with a recent longitudinal cohort study which reported significant parity-related differences in infant gut microbiome beta diversity up to 6 months of age among vaginally delivered infants.^36^ Our cross-sectional infant data suggest maternal factors associate with infant microbiome composition, but longitudinal sampling could reveal whether specific maternal subcommunities or temporal patterns influence infant microbiome assembly.

Our study has several limitations. Samples were collected using toilet paper wipes rather than direct stool collection, which introduced vaginal microbes into our samples. This sampling method was selected to maximize participant compliance across four collection timepoints. We applied conservative taxonomic filtering to remove known vaginal genera prior to analysis; notably, residual vaginal taxa segregated into a single subcommunity (SC3), supporting the effectiveness of our approach and enabling its exclusion from downstream analyzes. Additionally, our sample size (*n* = 52) limited power to detect subtle interactions, and missing samples at some time points reduced trajectory completeness. We were not able to address pregnancy-associated shifts in the gut microbiota that may occur earlier than 10 weeks of gestation, as participants were recruited during the first trimester of pregnancy. We also did not measure circulating metabolites, which would help determine whether microbiome stability in higher pBMI women corresponds to reduced metabolic adaptation.

In conclusion, pregnancy reshapes the balance among stable microbial subcommunities and parity modulates this process through what appears to be ecological memory, potentially mediated by host mucosal immune priming. These maternal dynamics may have intergenerational implications through their influence on infant gut colonization. Future studies integrating mucosal immune profiling, maternal metabolomics, and longitudinal infant sampling could clarify the mechanisms linking maternal microbiome trajectories to both maternal metabolic health and infant gut colonization outcomes.

## Supplementary Material

Supplementary Material SI_Kennedyetal_2026

Supplementary_Figures.docx Supplemental Material

KGMI_S_2690907.docx Supplemental Material
