# Supplementary material for: Longitudinal modelling of microbiome subcommunities reveals parity-dependent dynamics during pregnancy and postpartum
Source: Gut Microbes. 2026 Jul 15;18(1):2690907. doi: 10.1080/19490976.2026.2690907 (PMC13378715; doi:10.1080/19490976.2026.2690907)
Supplement: KGMI_S_2690907.docx — Supplemental Material [file KGMI_A_2690907_SM0703.docx]

**Table S1. Participant characteristics by intervention group**

| **Characteristic** | **Control** | **Intervention** | **p** |
| --- | --- | --- | --- |
| **n** | 25 | 27 |  |
| **Age** | 33.7 (4.4) | 31.9 (3.9) | 0.2¹ |
| **Primiparous** | 15 (60%) | 17 (63%) | 0.8² |
| **Primigravida** | 12 (48%) | 14 (52%) | 0.8² |
| **pBMI** | 23.8 (4.3) | 24.1 (4.1) | 0.5¹ |
| **GWG (kg/week)** | 0.47 (0.14) | 0.48 (0.15) | 1.0¹ |
| **Length of gestation (days)** | 278 (7) | 280 (7) | 0.3¹ |
| **Delivery mode = vaginal** | 19 (79%) | 18 (67%) | 0.3² |
| **Infant sex = female** | 12 (48%) | 12 (44%) | 0.8² |
| **Birth weight (g)** | 3605 (3350, 3720) | 3405 (3288, 3822) | 1.0¹ |
| **Birth length (cm)** | 51 (50, 53) | 52 (51, 54) | 0.2¹ |
| ¹ Wilcoxon rank sum exact test | | | |
| ² Pearson's Chi-squared test | | | |
| ³ Fisher's exact test | | | |
| Mean (SD); n (%); Median (Q1, Q3) | | | |

**Table S2. Agreement between TALDA and standard LDA topics**

| **Standard LDA topic** | **TALDA topic** | **Gamma concordance (Spearman ρ)** | **Beta Concordance (Cosine Similarity)** |
| --- | --- | --- | --- |
| **1** | 3 | 0.983862 | 0.999103 |
| **2** | 6 | 0.926822 | 0.99703 |
| **3** | 7 | 0.89044 | 0.99153 |
| **4** | 4 | 0.724219 | 0.976092 |
| **5** | 1 | 0.952621 | 0.998951 |
| **6** | 2 | 0.772871 | 0.96506 |
| **7** | 5 | 0.874818 | 0.988224 |
| Topics were matched across solutions using a greedy algorithm: for each standard LDA topic in sequence, the unmatched TALDA topic with the highest Spearman ρ in topic proportion (gamma) space was assigned. Beta cosine similarity was computed post-hoc for each matched pair. | | | |

**Table S3. Sensitivity of TALDA to exponential decay value**

| **Topic** | | **Beta matrix stability  (cosine similarity)** | | | **Gamma concordance  (Spearman ρ)** | | |
| --- | --- | --- | --- | --- | --- | --- | --- |
|  |  | **λ = 0.5 vs 0.75** | **λ = 0.5 vs 0.9** | **λ = 0.75 vs 0.9** | **λ = 0.5 vs 0.75** | **λ = 0.5 vs 0.9** | **λ = 0.75 vs 0.9** |
| **1** |  | 0.998 | 0.996 | 1.000 | 0.948 | 0.947 | 0.990 |
| **2** |  | 0.965 | 0.970 | 0.998 | 0.622 | 0.651 | 0.965 |
| **3** |  | 0.999 | 0.999 | 0.999 | 0.992 | 0.992 | 0.996 |
| **4** |  | 0.867 | 0.880 | 0.997 | 0.519 | 0.540 | 0.973 |
| **5** |  | 0.972 | 0.968 | 0.997 | 0.801 | 0.791 | 0.911 |
| **6** |  | 0.985 | 0.991 | 0.998 | 0.825 | 0.750 | 0.905 |
| **7** |  | 0.993 | 0.992 | 0.999 | 0.858 | 0.805 | 0.951 |

*Three decay values were compared: λ = 0.5 (aggressive down-weighting), λ = 0.75 (selected value; moderate down-weighting), and λ = 0.9 (mild down-weighting). Pairwise cosine similarity between topic definitions (beta matrices) for matched topics. Pairwise Spearman ρ between topic proportion (gamma) vectors for matched topics.*

**Supplemental Figure 1. Delivery mode, solid food introduction, and probiotic intake influence subcommunity composition in breastfed infants at 6 months of age** Mean (points) ± s.e.m. (error bars) of subcommunity (topic) probabilities in breastfed infants at 6 months. Left panels: Breastfed infants consuming solid foods, stratified by probiotic intake (No probiotics, Probiotics). Right panels: Breastfed infants not consuming probiotics, stratified by solid food introduction (No solids, Solids). Semi-transparent points/error bars (■, ●) show delivery mode-specific means (CS, vaginal) in left panels and solid food-specific means (No solids, Solids) in right panels, overlaid with opaque points/error bars indicating the panel mean across groups for each subcommunity.
